# Correction: PCGF6-PRC1 suppresses premature differentiation of mouse embryonic stem cells by regulating germ cell-related genes

**DOI:** 10.7554/eLife.27970

**Published:** 2017-04-25

**Authors:** Mitsuhiro Endoh, Takaho A Endo, Jun Shinga, Katsuhiko Hayashi, Anca Farcas, Kit-Wan Ma, Shinsuke Ito, Jafar Sharif, Tamie Endoh, Naoko Onaga, Manabu Nakayama, Tomoyuki Ishikura, Osamu Masui, Benedikt M Kessler, Toshio Suda, Osamu Ohara, Akihiko Okuda, Robert J Klose, Haruhiko Koseki

Endoh M, Endo TA, Shinga J, Hayashi J, Farcas A, Ma K-W, Ito S, Sharif J, Endoh T, Onaga N, Nakayama M, Ishikura T, Masui O, Kessler BM, Suda T, Ohara O, Okuda A, Klose R, Koseki H. 2017. PCGF6-PRC1 suppresses premature differentiation of mouse embryonic stem cells by regulating germ cell-related genes. *eLife* **6**:e21064. doi: 10.7554/eLife.21064.

Published 17, March 2017

We would like to thank Albrecht Müller and Matthias Becker, who pointed out the omission of the citation in the original article.

In the published article, the article shown below should be cited in the Results.

Zdzieblo D, Li X, Lin Q, Zenke M, Illich DJ, Becker M, Müller AM. 2014. Pcgf6, a polycomb group protein, regulates mesodermal lineage differentiation in murine ESCs and functions in iPS reprogramming. *Stem Cells* **32**:3112-3125. doi: 10.1002/stem.1826.

In the first sentence of the chapter “PCGF6 represses germ cell-related genes via RING1B catalytic activy”, the text ‘PCGF6-PRC1 target genes are enriched in meiosis- and germ cell-related genes’ has been replaced with ‘PCGF6-PRC1 target genes are enriched in meiosis- and germ cell-related genes as suggested previously (Zdzieblo et al., 2014)’.

In the second sentence of the chapter “PCGF6-PRC1 is required for maintenance of ESCs and epiLCs in an undifferentiated state”, the text “in the presence of three inhibitors (3i: SU5402 for FGFR, PD184352 for ERK, and CHIR99021 for GSK3) and fetal bovine serum (Figure 5A,B)’ has been replaced with ‘in the presence of three inhibitors (3i: SU5402 for FGFR, PD184352 for ERK, and CHIR99021 for GSK3) and fetal bovine serum as suggested previously (Zdzieblo et al., 2014) (Figure 5A,B).

We apologize for any confusion that may have occurred.

The article has been corrected accordingly.

