# Supplementary figures and images for: PCGF6-PRC1 suppresses premature differentiation of mouse embryonic stem cells by regulating germ cell-related genes
Source: eLife. 2017 Mar 17;6:e21064. doi: 10.7554/eLife.21064 (PMC5375644; doi:10.7554/eLife.21064)

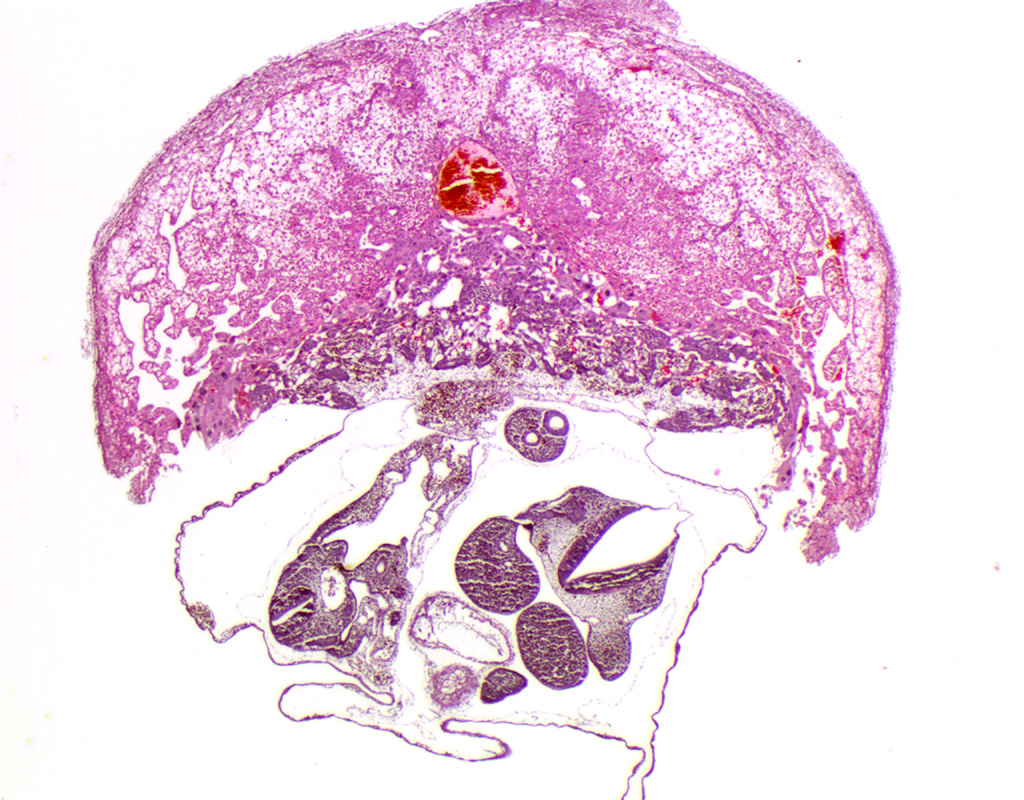

Supplement: Figure 6—source data 1. — DOI: http://dx.doi.org/10.7554/eLife.21064.014 [file elife-21064-fig6-data1.zip › MBLR_PLACENTAE/MBLR-het.F.10dpc.#2600.1.jpg]

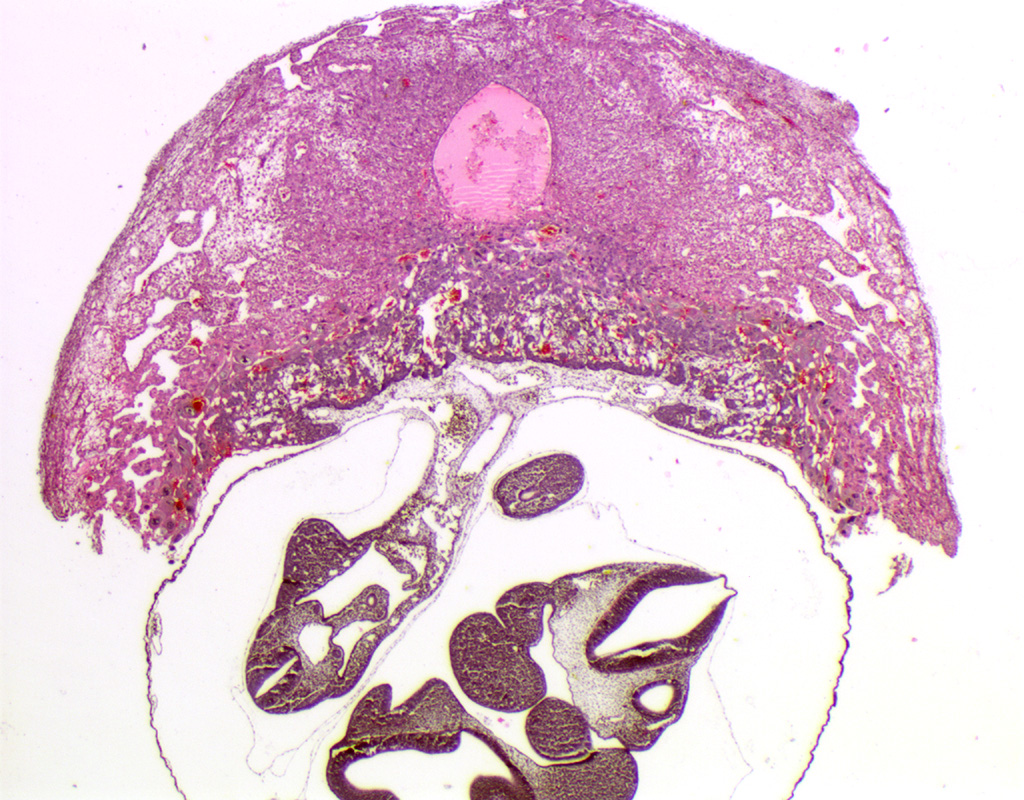

Supplement: Figure 6—source data 1. — DOI: http://dx.doi.org/10.7554/eLife.21064.014 [file elife-21064-fig6-data1.zip › MBLR_PLACENTAE/MBLR-het.F.10dpc.#2612.1.jpg]

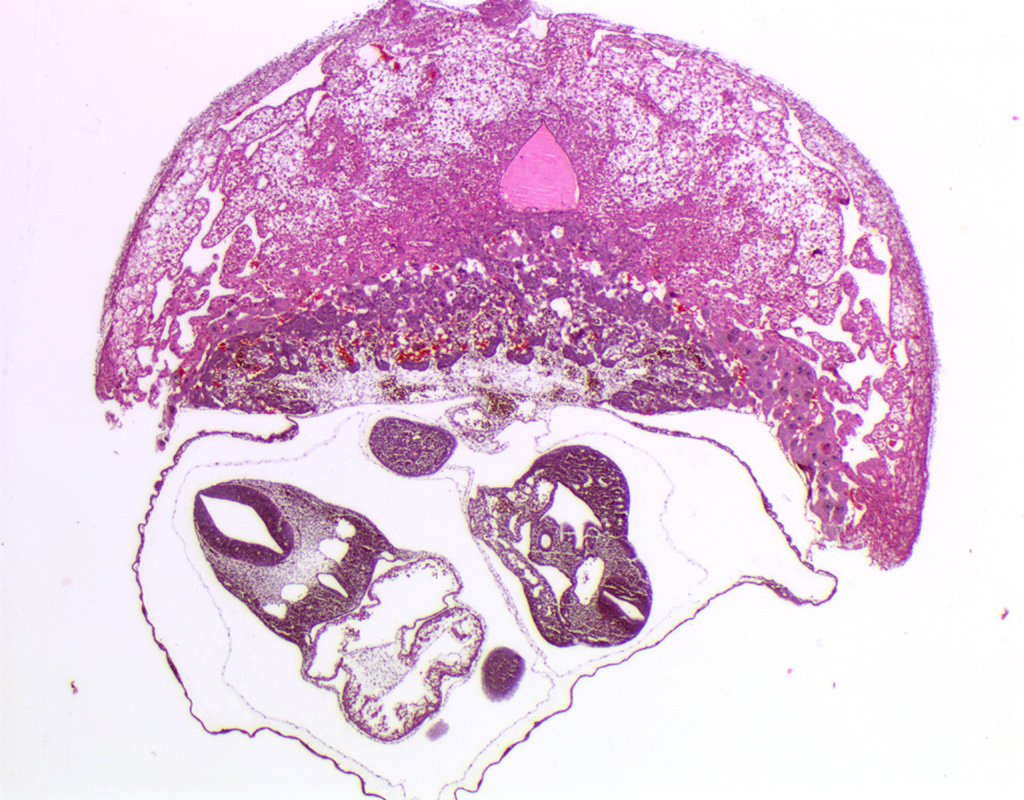

Supplement: Figure 6—source data 1. — DOI: http://dx.doi.org/10.7554/eLife.21064.014 [file elife-21064-fig6-data1.zip › MBLR_PLACENTAE/MBLR-het.M.10dpc.#2597.1.jpg]

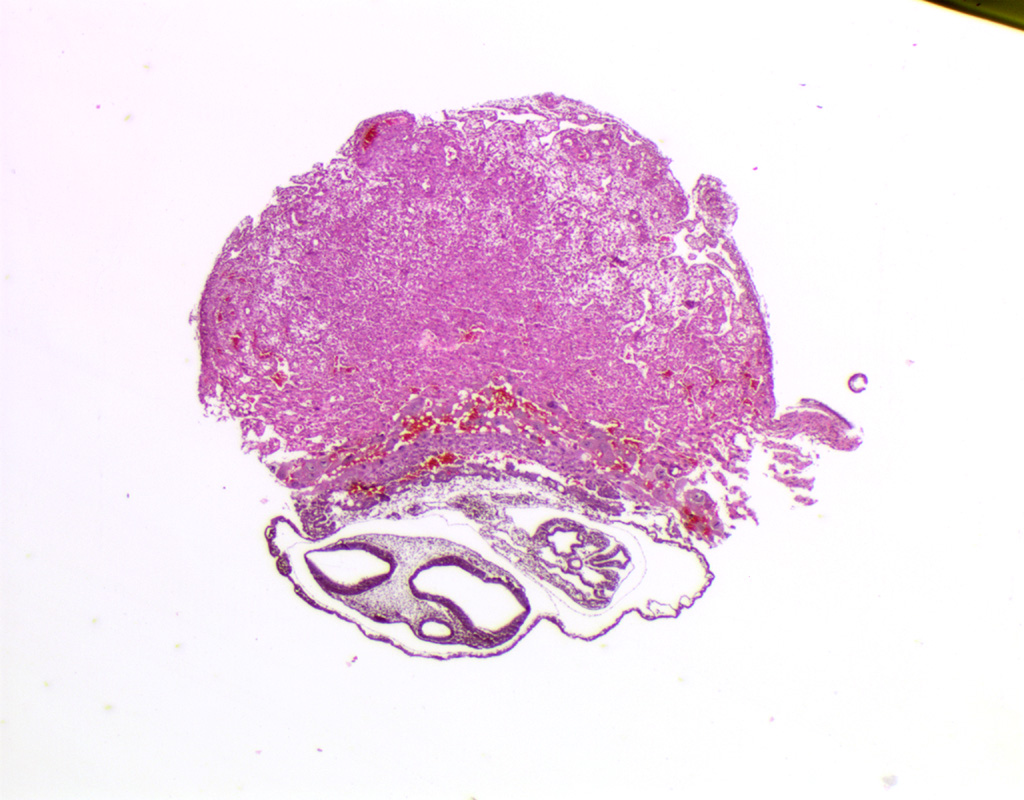

Supplement: Figure 6—source data 1. — DOI: http://dx.doi.org/10.7554/eLife.21064.014 [file elife-21064-fig6-data1.zip › MBLR_PLACENTAE/MBLR-het.M.10dpc.#2603.1.jpg]

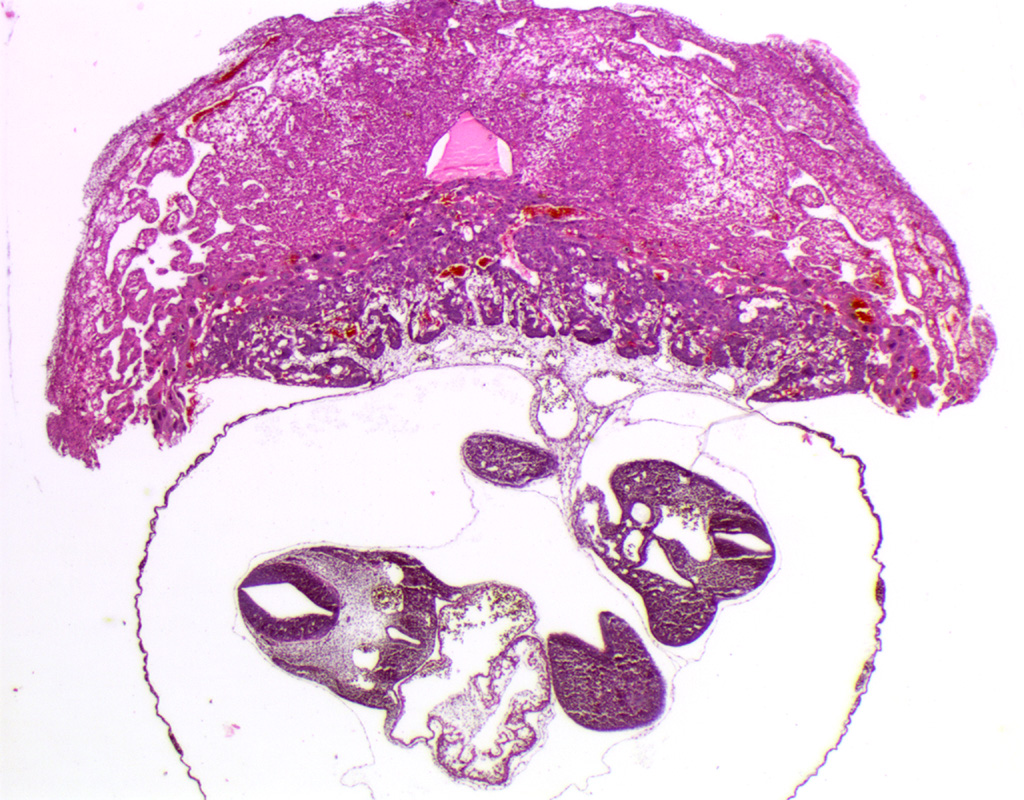

Supplement: Figure 6—source data 1. — DOI: http://dx.doi.org/10.7554/eLife.21064.014 [file elife-21064-fig6-data1.zip › MBLR_PLACENTAE/MBLR-het.M.10dpc.#2613.1.jpg]

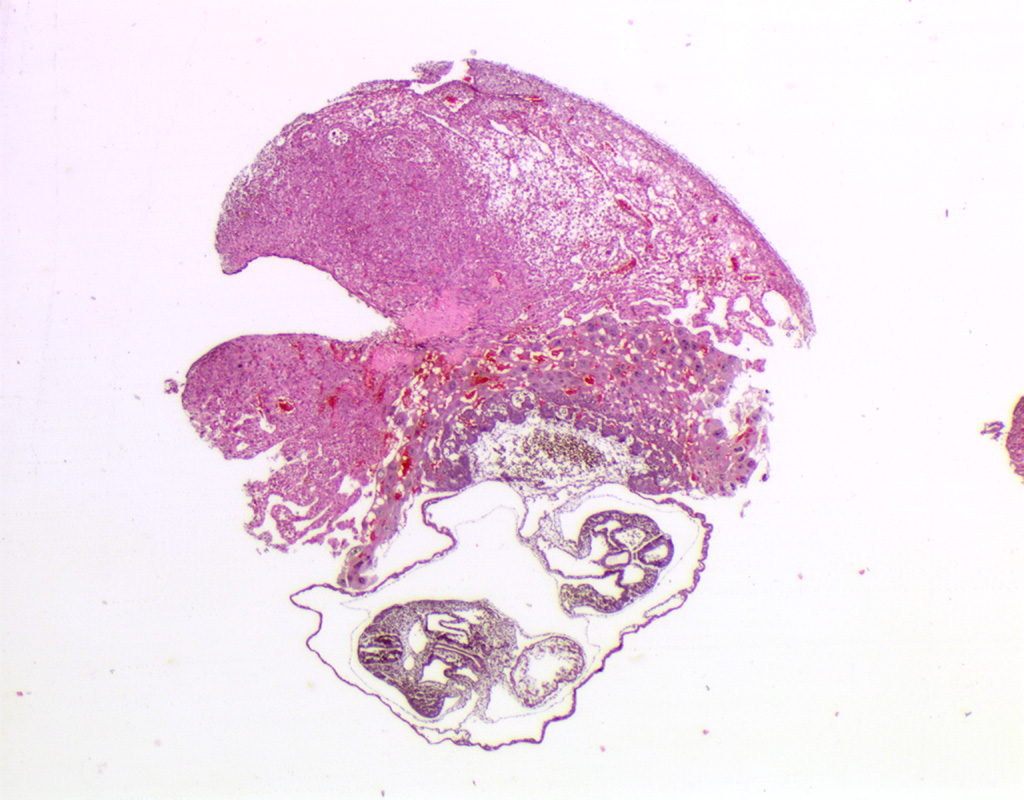

Supplement: Figure 6—source data 1. — DOI: http://dx.doi.org/10.7554/eLife.21064.014 [file elife-21064-fig6-data1.zip › MBLR_PLACENTAE/MBLR-ko.F.10dpc.#2604.1.jpg]

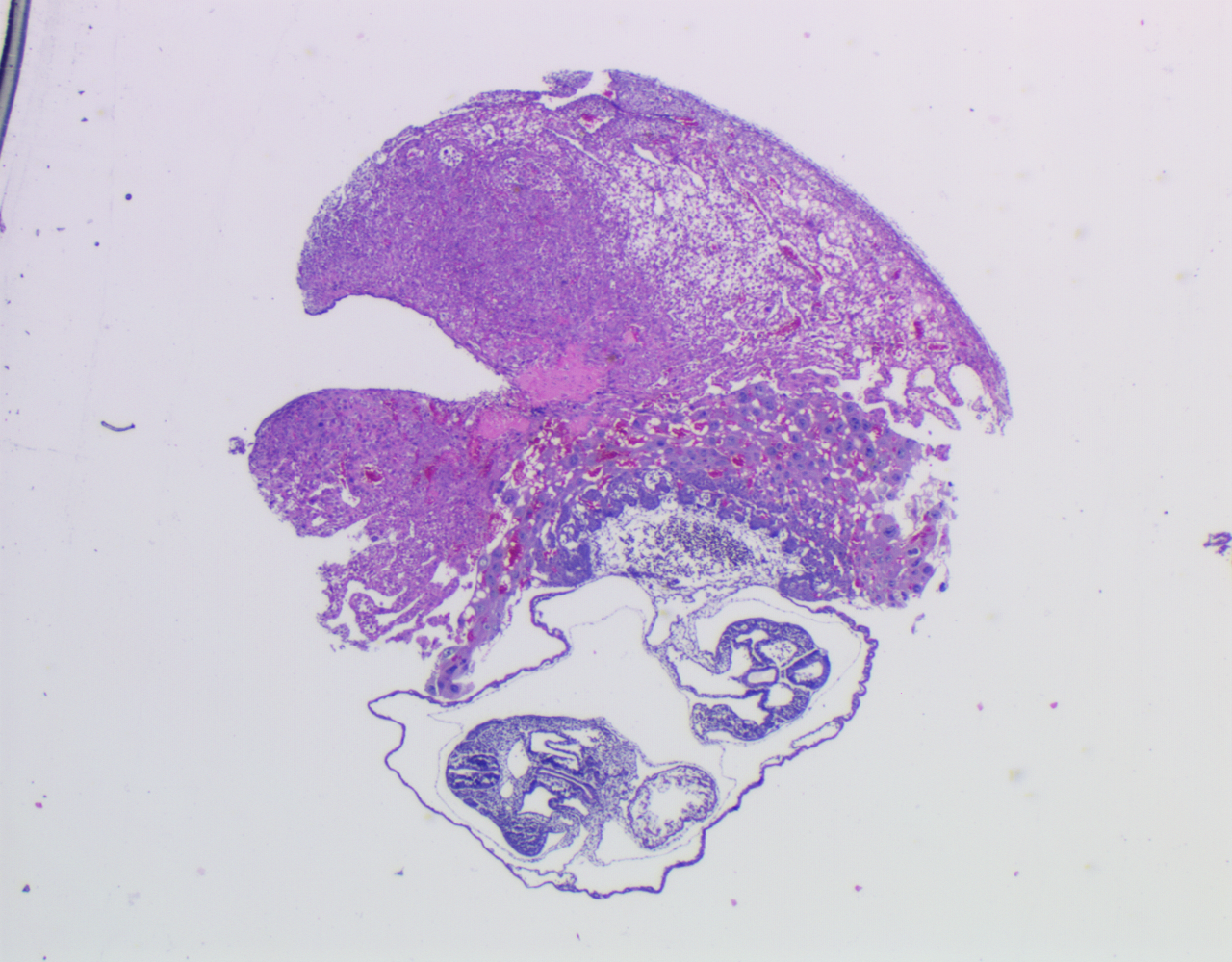

Supplement: Figure 6—source data 1. — DOI: http://dx.doi.org/10.7554/eLife.21064.014 [file elife-21064-fig6-data1.zip › MBLR_PLACENTAE/MBLR-ko.F.10dpc.#2604.tiff]

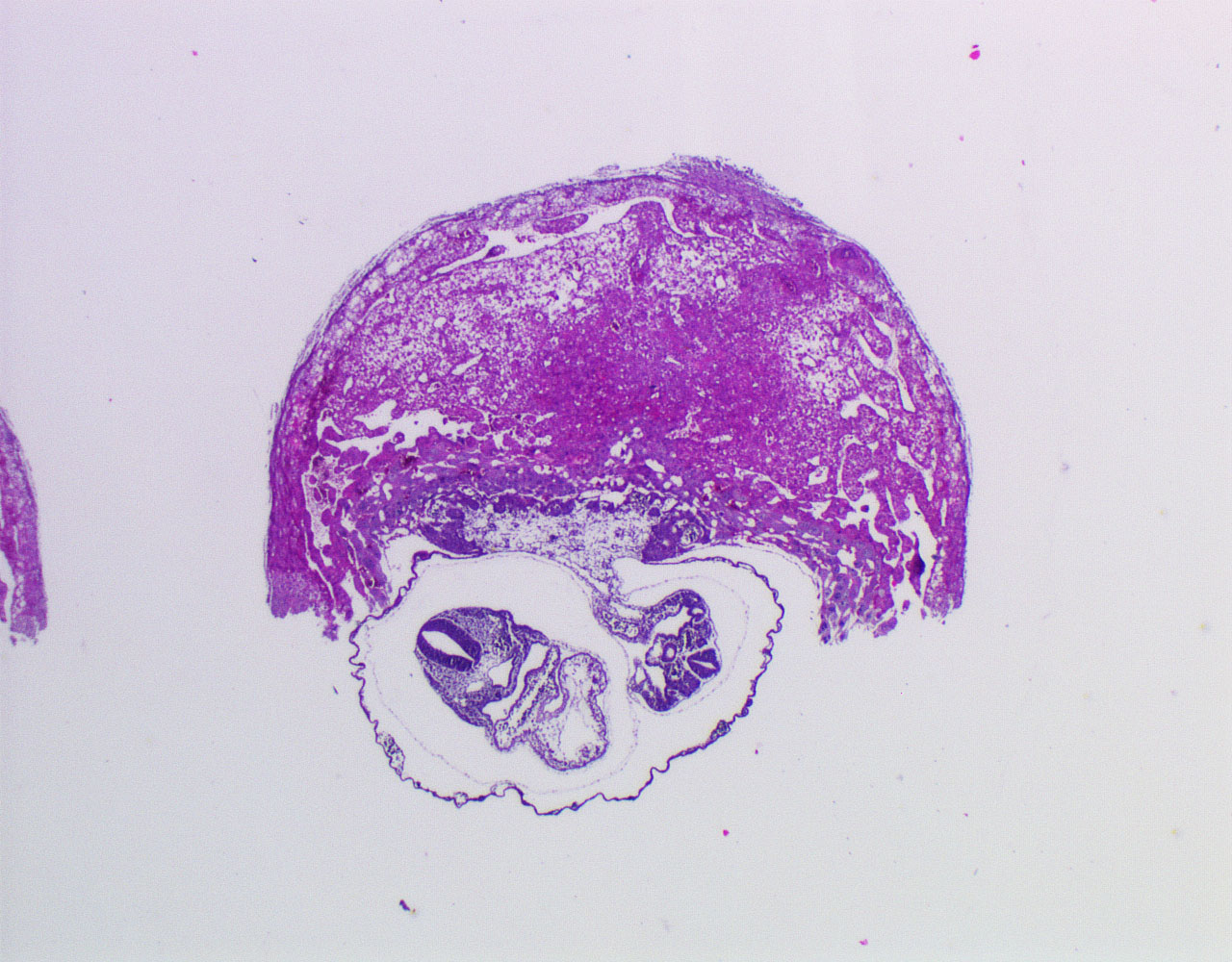

Supplement: Figure 6—source data 1. — DOI: http://dx.doi.org/10.7554/eLife.21064.014 [file elife-21064-fig6-data1.zip › MBLR_PLACENTAE/MBLR-ko.F.10dpc.#2901.1.jpg]

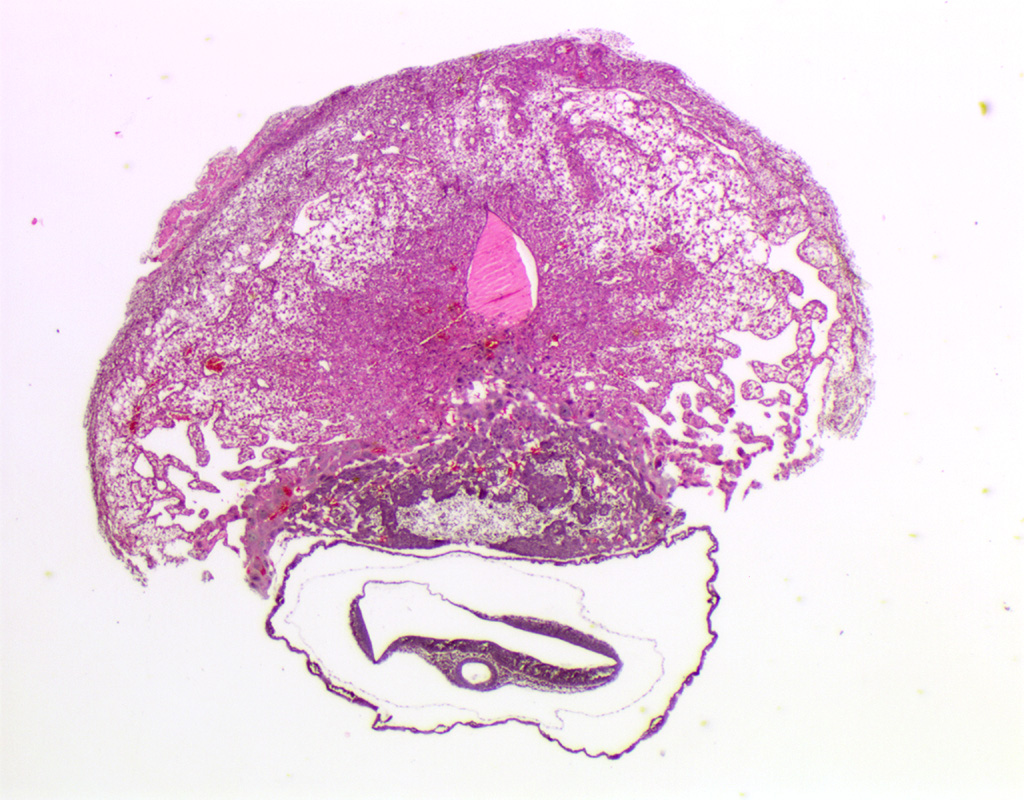

Supplement: Figure 6—source data 1. — DOI: http://dx.doi.org/10.7554/eLife.21064.014 [file elife-21064-fig6-data1.zip › MBLR_PLACENTAE/MBLR-ko.M.10dpc.#2596.1.jpg]

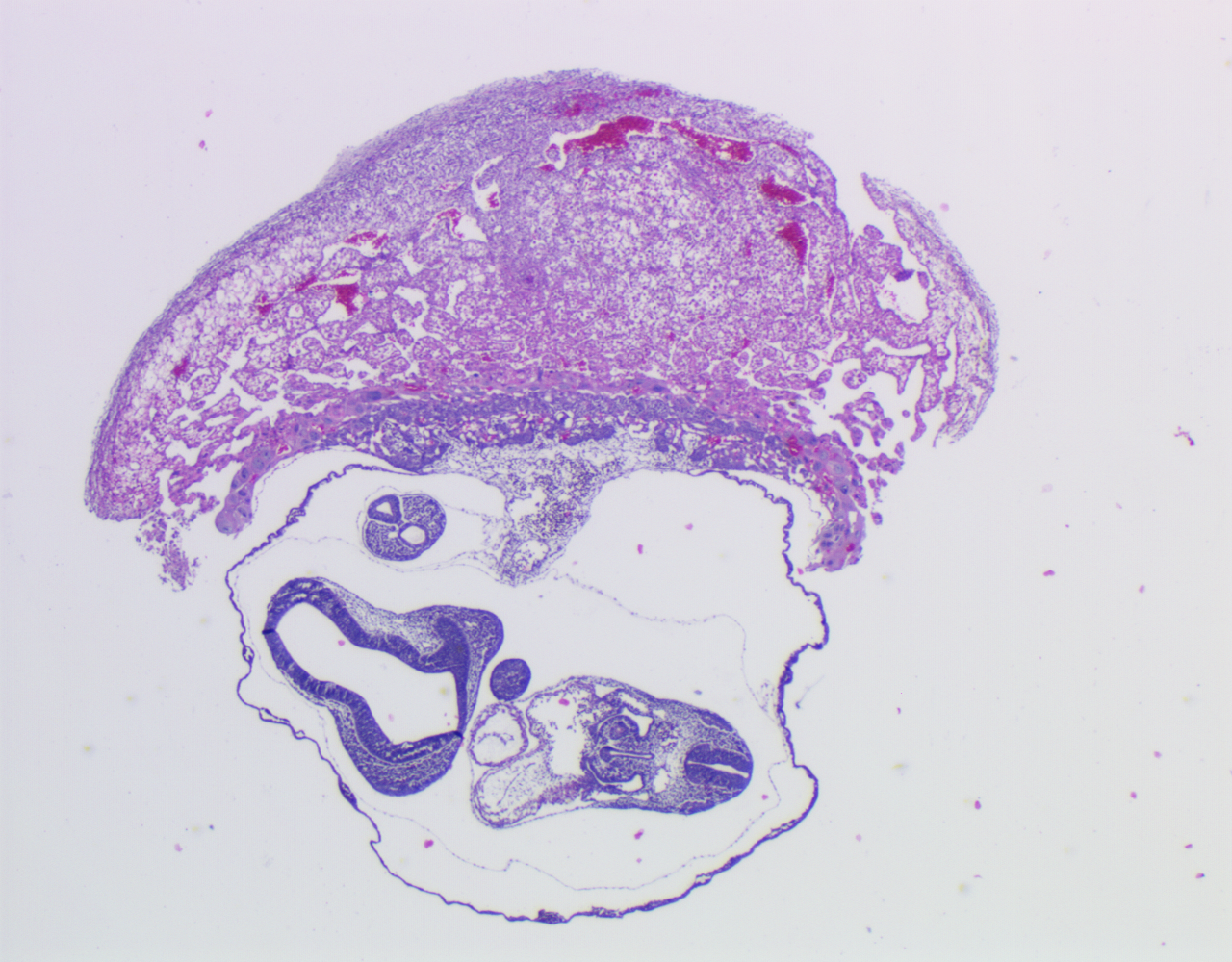

Supplement: Figure 6—source data 1. — DOI: http://dx.doi.org/10.7554/eLife.21064.014 [file elife-21064-fig6-data1.zip › MBLR_PLACENTAE/MBLR-ko.M.10dpc.#2596.tiff]

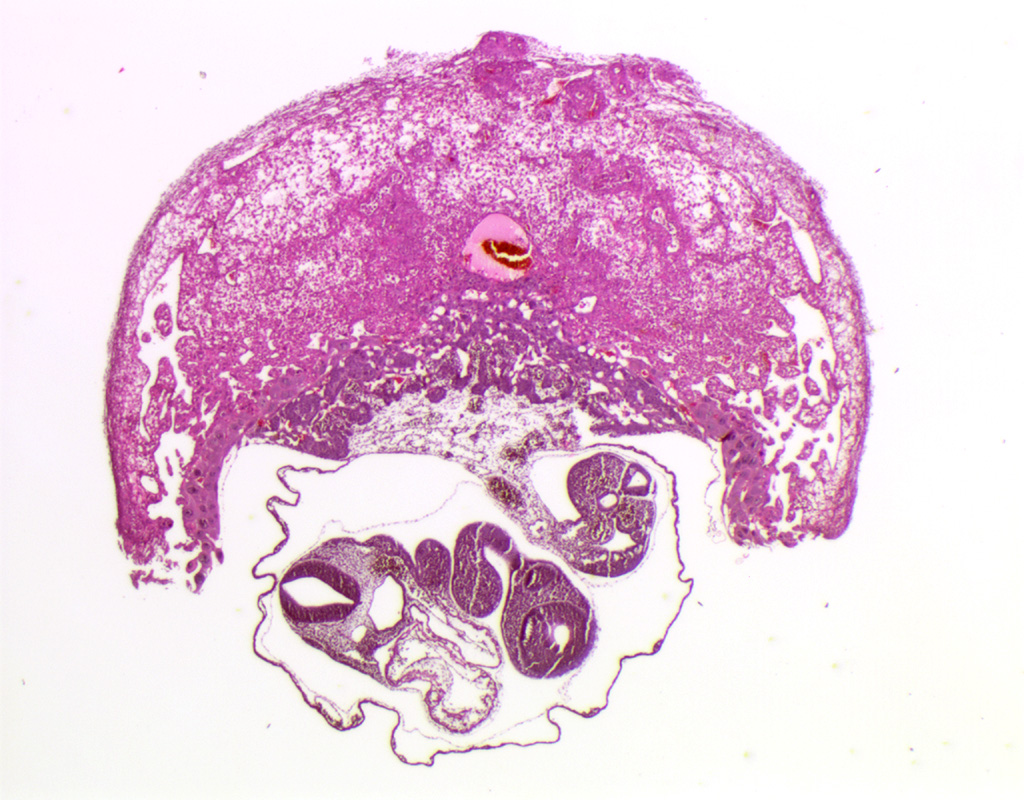

Supplement: Figure 6—source data 1. — DOI: http://dx.doi.org/10.7554/eLife.21064.014 [file elife-21064-fig6-data1.zip › MBLR_PLACENTAE/MBLR-ko.M.10dpc.#2598.1.jpg]

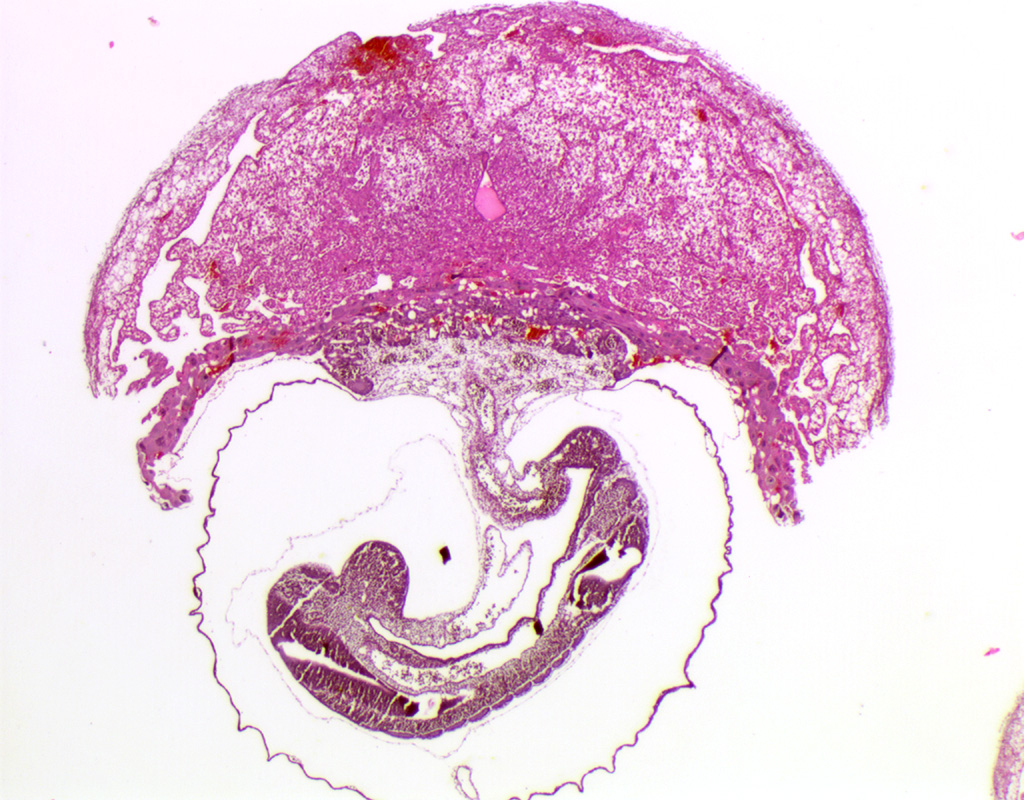

Supplement: Figure 6—source data 1. — DOI: http://dx.doi.org/10.7554/eLife.21064.014 [file elife-21064-fig6-data1.zip › MBLR_PLACENTAE/MBLR-ko.M.10dpc.#2599.1.jpg]

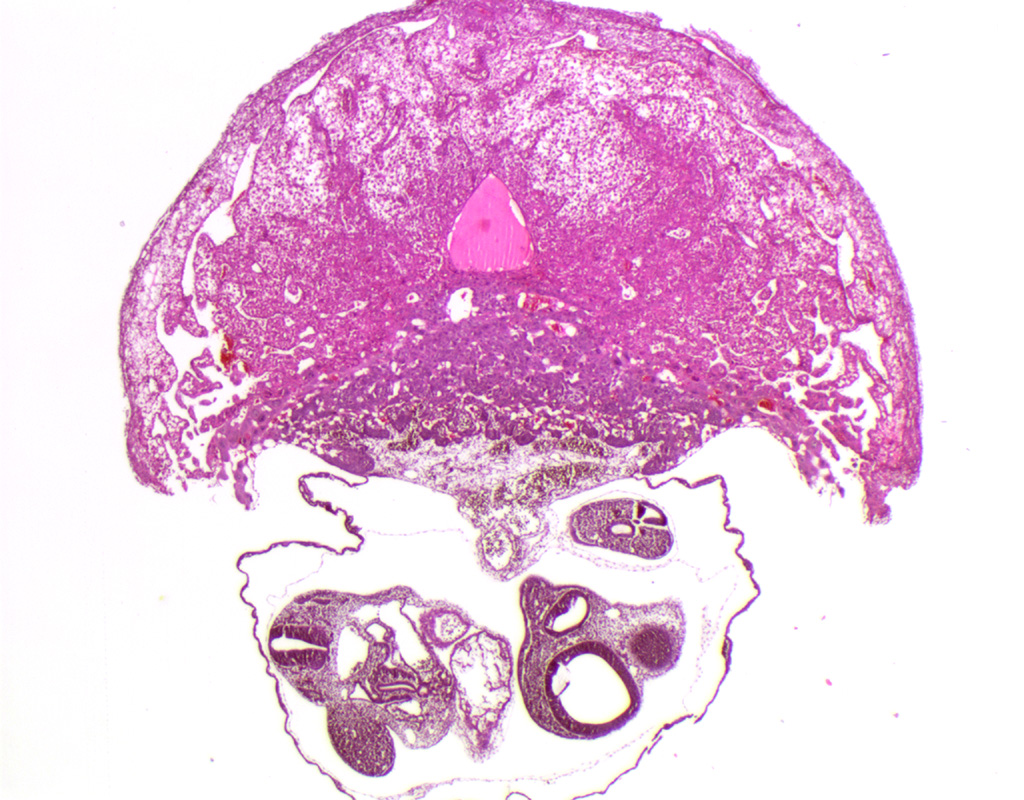

Supplement: Figure 6—source data 1. — DOI: http://dx.doi.org/10.7554/eLife.21064.014 [file elife-21064-fig6-data1.zip › MBLR_PLACENTAE/MBLR-ko.M.10dpc.#2601.1.jpg]

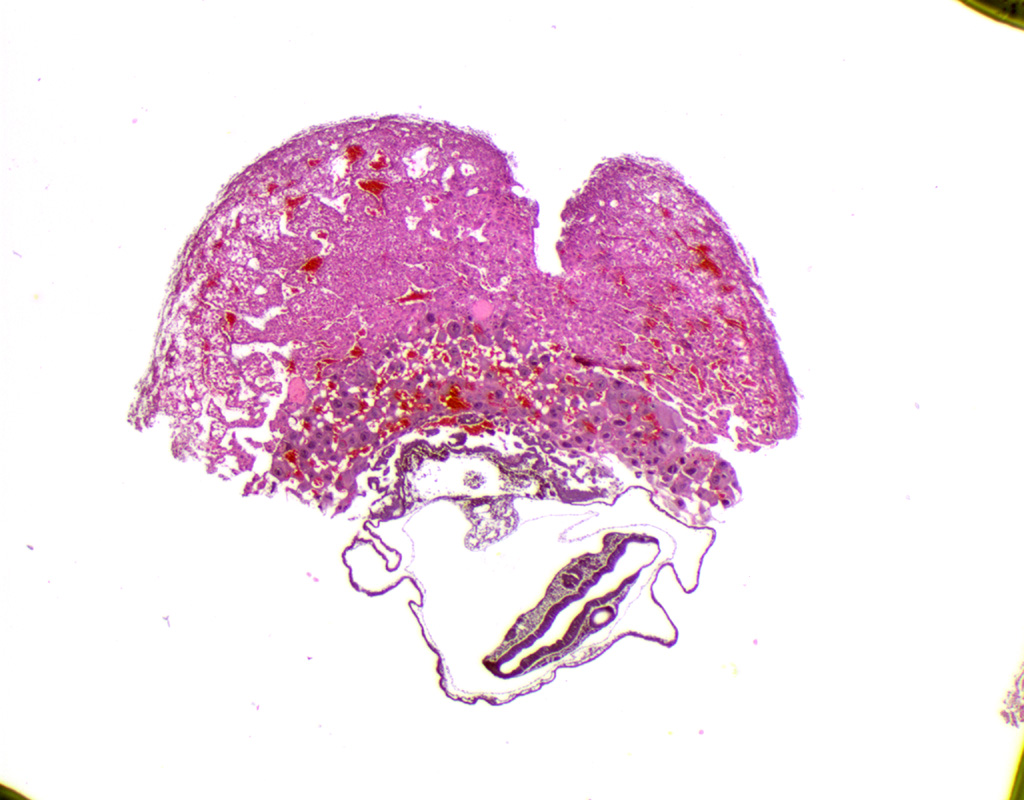

Supplement: Figure 6—source data 1. — DOI: http://dx.doi.org/10.7554/eLife.21064.014 [file elife-21064-fig6-data1.zip › MBLR_PLACENTAE/MBLR-ko.M.10dpc.#2605.1.jpg]

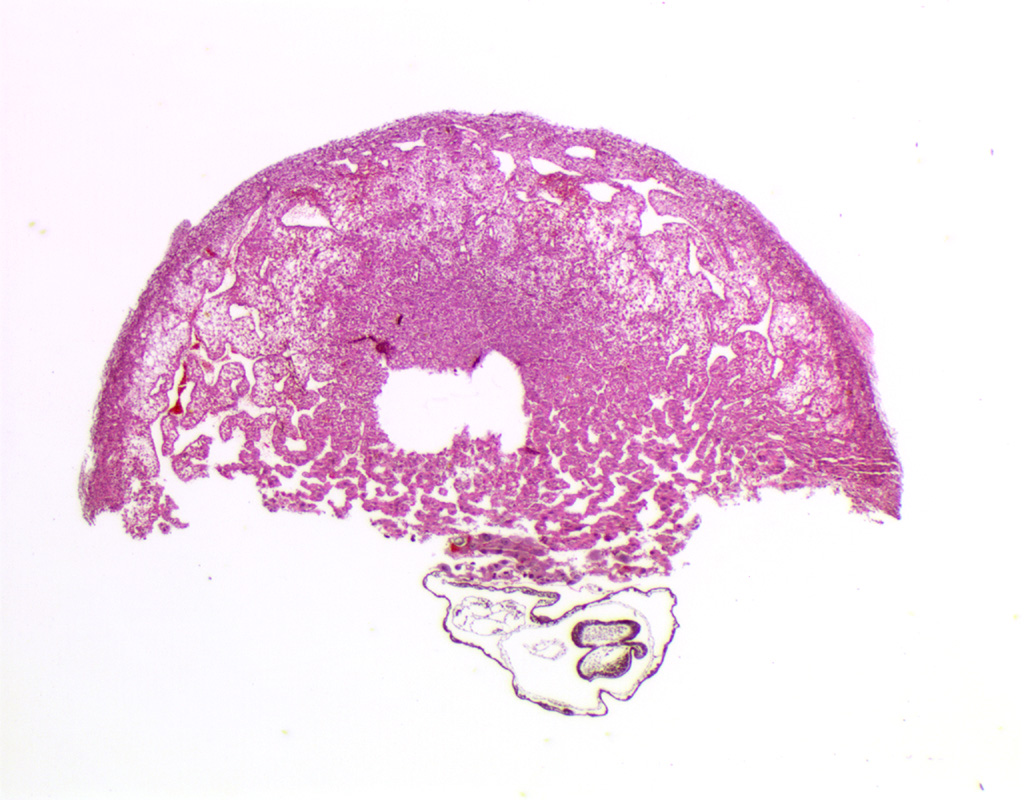

Supplement: Figure 6—source data 1. — DOI: http://dx.doi.org/10.7554/eLife.21064.014 [file elife-21064-fig6-data1.zip › MBLR_PLACENTAE/MBLR-ko.M.10dpc.#2609.1.jpg]

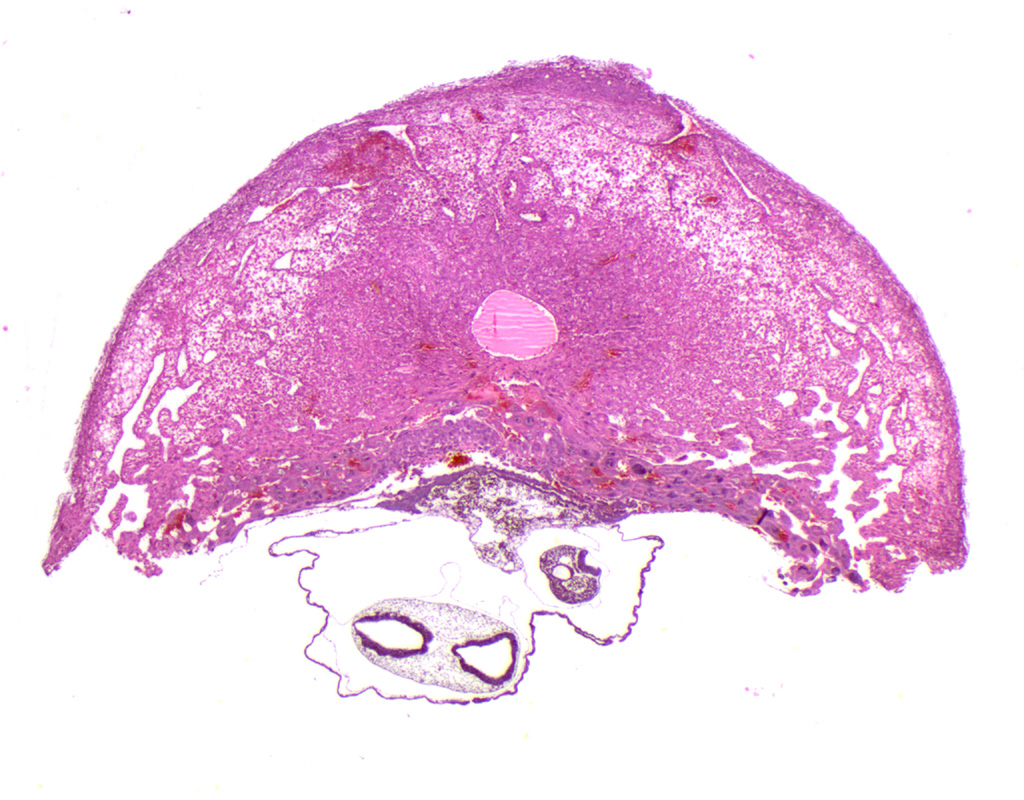

Supplement: Figure 6—source data 1. — DOI: http://dx.doi.org/10.7554/eLife.21064.014 [file elife-21064-fig6-data1.zip › MBLR_PLACENTAE/MBLR-ko.M.10dpc.#2611.1.jpg]

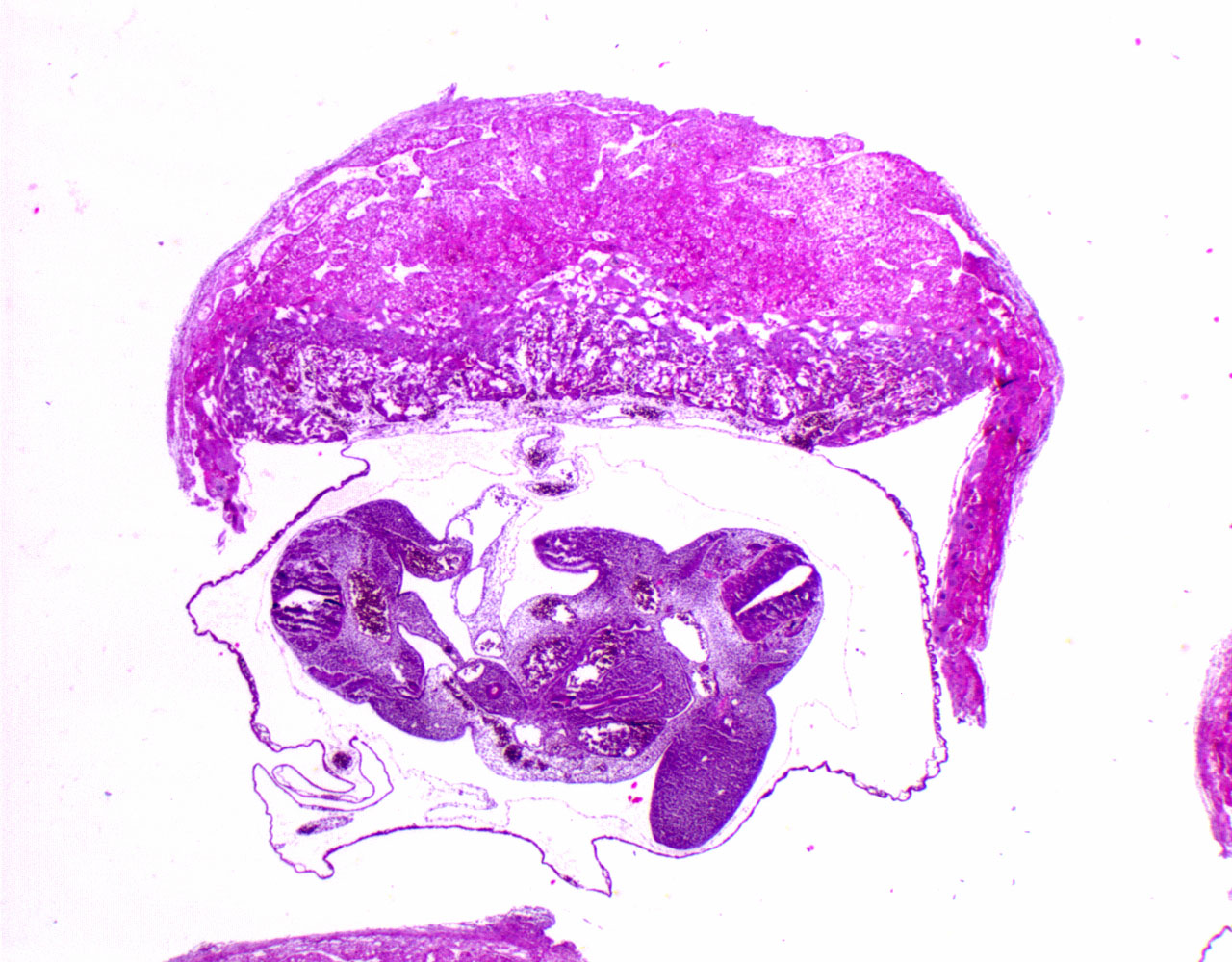

Supplement: Figure 6—source data 1. — DOI: http://dx.doi.org/10.7554/eLife.21064.014 [file elife-21064-fig6-data1.zip › MBLR_PLACENTAE/MBLR-wt.F.10dpc.#2922.1.jpg]
