# Supplementary material for: PCGF6-PRC1 suppresses premature differentiation of mouse embryonic stem cells by regulating germ cell-related genes
Source: eLife. 2017 Mar 17;6:e21064. doi: 10.7554/eLife.21064 (PMC5375644; doi:10.7554/eLife.21064)
Supplement: Supplementary file 1. — DOI: http://dx.doi.org/10.7554/eLife.21064.017 [file elife-21064-supp1.docx]

**Supplementary File 1**

**The sequences of primers used in quantitative ChIP-PCR and RT-PCR**

[For ChIP-qPCR]

*Ddx4* Forward primer: GAGAGAGAAACGGGATGTCG

*Ddx4* Reverse primer: CGGGGACAACAAATAGCATC

*Tdrd1* Forward primer: CGCACGTGGGAGTAAACTG

*Tdrd1* Reverse primer: CTGGTTTCTGAAGCTGACCA

*Mael* Forward primer: GGAATCCAGTTTCAGGCTGT

*Mael* Reverse primer: GCTCCCGCCCTTAGTAACC

*Zic1* Forward primer: CAAACCTCAGGAACCAGGAA

*Zic1* Reverse primer: TGGTGTCTTTCACAGCCTCA

*Stag3* Forward primer: GACACAGATGGAGCAGCAAA

*Stag3* Reverse primer: AGGGGACACGCTCCTATTCT

*Spo11* Forward primer: GTTCTTCGACGCCCTGGAT

*Spo11* Reverse primer: CTGCCGAGCTGCTGTACCTA

*Hoxd11* Forward primer: CACTCTTGTCCCTGGTGTCA

*Hoxd11* Reverse primer: CTGGGAGCTTGTTGCTTCTT

*Syce1* Forward primer: GCAGTGTCTAACAGCCTGGA

*Syce1* Reverse primer: CCCTTCAGCCACTACCTCTG

*Igf2r* Forward primer: TTGTCAGGCCTCGAGTAGGTA

*Igf2r* Reverse primer: GTCCTCGTTCAGGTGCTCTC

*Agpat5* Forward primer: CCACCACCTCACTTCTTCCC

*Agpat5* Reverse primer: TGTTCTGATTGGCTCGGTCC

*Rpp25* Forward primer: AACTCTGGCCCTTTTGCAGA

*Rpp25* Reverse primer: GCCAGTAGGTTGCGGATCTT

*Eif5a2* Forward primer: ATAGTCGTCCCCAAGGTCAA

*Eif5a2* Reverse primer: CTGCGGATTAGTGCACTTCC

*Chr8 intergenic* Forward primer: AAGGGGCCTCTGCTTAAAAA

*Chr8 intergenic* Reverse primer: AGAGCTCCATGGCAGGTAGA

*Chr6 intergenic* Forward primer: CCCCTTTCTGAAGCACTCTG

*Chr6 intergenic* Reverse primer: TAAGGCGTCATTTCCCAAAG

*Bmp7* Forward primer: CGAGCCAGCAAGACTTATCC

*Bmp7* Reverse primer: CTGTGGTCCACCTTTCCATT

[For RT-qPCR]

*Hoxd11* Forward primer: CTCCAACTCTCTCGGATGCT

*Hoxd11* Reverse primer: CAGACGGTCCCTGTTCAGTT

*Zic1* Forward primer: CTTTTCCCTGCCCGTTTC

*Zic1* Reverse primer: CTCGAACTCGCACTTGAAGG

*Pou5f1* Forward primer: AGAGGGAACCTCCTCTGAGC

*Pou5f1* Reverse primer: CCAAGGTGATCCTCTTCTGC

*Nanog* Forward primer: CACCCACCCATGCTAGTCTT

*Nanog* Reverse primer: ACCCTCAAACTCCTGGTCCT

*Gapdh* Forward primer: ACCACAGTCCATGCCATCAC

*Gapdh* Reverse primer: TCCACCACCCTGTTGCTGTA

*Dazl* Forward primer: GCACTCAGTCTTCATCAGCAAC

*Dazl* Reverse primer: CTGGTGAACTTGGATAAGGAGG

*Mael* Forward primer: CGAGGATTTCGATTCCATTGCC

*Mael* Reverse primer: GGCTCTATCATCAGACTTGCAGT

*Piwil2* Forward primer: GCTGCTCCTGATCACTTCGT

*Piwil2* Reverse primer: CCAGGCCAATTCCAGTACAT

*Zfp352* Forward primer: GCTTATAGAGAAGACCAGCTAC

*Zfp352* Reverse primer: CATCTCCATATAGACTTCACAG

*Tcstv3* Forward primer: ACCAGCTGAAACATCCATCC

*Tcstv3* Reverse primer: CCATGGATCCCTGAAGGTAA

*MuERV* Forward primer: CTTCCATTCACAGCTGCGACTG

*MuERV* Reverse primer: CTAGAACCACTCCTGGTACCAAC

*Gata6* Forward primer: GACGGCACCGGTCATTACC

*Gata6* Reverse primer: ACAGTTGGCACAGGACAGTCC

*Syce1* Forward primer: GGGGTACAGATCCTTGCTCA

*Syce1* Reverse primer: AGCAAGAGACAGGGAAAGCA

*Tdrkh* Forward primer: TCCAGAGACGTCCATGTTTG

*Tdrkh* Reverse primer: TGCTGGGTCATCTCACTGAC

*Stag3* Forward primer: CACCTACTGCCACTCCCTTC

*Stag3* Reverse primer: TCAGCTGAAGGCTTTCTTCC
